# Editorial: Adverse Vaccine Reactions

**DOI:** 10.3389/falgy.2022.948380

**Published:** 2022-06-14

**Authors:** Ingrid Terreehorst

**Affiliations:** Department of ENT, Amsterdam University Medical Center, Amsterdam, Netherlands

**Keywords:** vaccine, COVID-19, PEG, anaphylactic reactions, side effects

Vaccines against infectious diseases are without doubt of great value in reducing the prevalence of for instance rubella and polio in children. In adult age, vaccination is useful in the prevention of severe comorbidity such as pneumonia in measles. Unfortunately, nowadays vaccination is discarded by certain groups that link vaccines to morbidities such as autism and auto immune disease despite lack of evidence. Nevertheless, since vaccines are mainly preventive in nature and have no immediate benefits, the acceptable rate of side effects is much lower than in for instance antibiotics, chemotherapy or biologicals that are used in the treatment of infections, malignancies and auto immune diseases respectively; serious side effects of vaccines will be even less acceptable, both in- and outside the medical community.

COVID-19 disease has had major impact on our society, not only in terms of morbidity and mortality, but also socially and economically. Therefore, when the first vaccines were presented in December 2020 against COVID-19, they were seen as a possible quick route out of the epidemic and back to normal life. Shortly after the first introduction, reports about anaphylaxis appeared and indicated a much higher prevalence than in the regular vaccines, causing growing concern about their safety. Moreover, there appeared to be side effects such as thrombosis (TTS), myocarditis and pericarditis. In this Research Topic, Adverse Vaccine Reaction all aspects of COVID-19 disease vaccine safety are addressed.

Soon after the first reports of anaphylaxis were published, several countries and Allergy societies developed guidelines to ensure safe procedures and to identify patients at risk before vaccination takes place (1). One of these guidelines is the Consensus statements on the Approach to COVID-19 Vaccine Allergy Safety in Hong Kong by Chiang et al.. They used the Delphi procedure to form consensus statements regarding identification of patients at risk, safety procedures of the vaccination process and preferred management during and after a reaction.

Although the incidence of anaphylaxis was first thought to be high, millions have received their first vaccinations as well as boosters and anaphylaxis rates are now thought to be around 5 per million vaccine doses (2), which is still higher than other vaccines such as Hib, HBV, TIV, Pertussis, Pneumococcal and IPV which rate between 0 and 2.89 per million doses. Only MMR has an anaphylaxis rate of 5.14 per million doses according to the EAACI paper by Nilsson et al. (3).

The usual procedure in allergic reactions is to try and identify the culprit by means of skin tests and/or specific IgE in serum. Stehlin et al. report on the predictive value of skin tests in suspected anaphylaxis after COVID-19 disease vaccination. Their study consists of two cohorts, a group with a high risk profile and a post vaccination group. All negative tested patients tolerated vaccination while positive tests could be confirmed with the basophil activation test, indicating good reliability of the skin test.

The amount of additives in the various vaccines is high and most of them are known to be innocent such as potassium chloride, sodium chloride and saccharose. Others on the other hand were already known as rare elicitors of anaphylaxis in perioperative anaphylaxis such as PEG and polysorbate 80 (4). McSweeney et al. describe a patient experiencing anaphylaxis after Pfizer/BioNTech COVID-19 disease vaccine, where PEG was confirmed to be the culprit allergen.

Although PEG is considered a potential culprit, there are many other ways of activating mast cells. Laisuan has reviewed the evidence for the various causes of anaphylactic reactions after COVID vaccination, including allergic and non-allergic pathways.

## Author Contributions

The author confirms being the sole contributor of this work and has approved it for publication.

## Conflict of Interest

The author declares that the research was conducted in the absence of any commercial or financial relationships that could be construed as a potential conflict of interest.

## Publisher's Note

All claims expressed in this article are solely those of the authors and do not necessarily represent those of their affiliated organizations, or those of the publisher, the editors and the reviewers. Any product that may be evaluated in this article, or claim that may be made by its manufacturer, is not guaranteed or endorsed by the publisher.
